# Nanobodies: new avenue to treat kidney disease

**DOI:** 10.1007/s00441-021-03479-8

**Published:** 2021-06-16

**Authors:** Nicola Wanner, Thomas Eden, Nastassia Liaukouskaya, Friedrich Koch-Nolte

**Affiliations:** 1 III. Department of Medicine, University Medical Center Hamburg-Eppendorf (UKE), Hamburg, Germany; 2 Institute of Immunology, University Medical Center Hamburg-Eppendorf (UKE), Hamburg, Germany

**Keywords:** Nanobodies, Renal diseases, Conventional antibodies

## Abstract

Current therapeutic options for renal diseases are limited, and the search for disease-specific treatments is ongoing. Nanobodies, single-domain antibodies with many advantages over conventional antibodies, provide flexible, easy-to-format biologicals with many possible applications. Here, we discuss the potential use of nanobodies for renal diseases.

## Nanobody advantages and formats

**Fig. 1:**
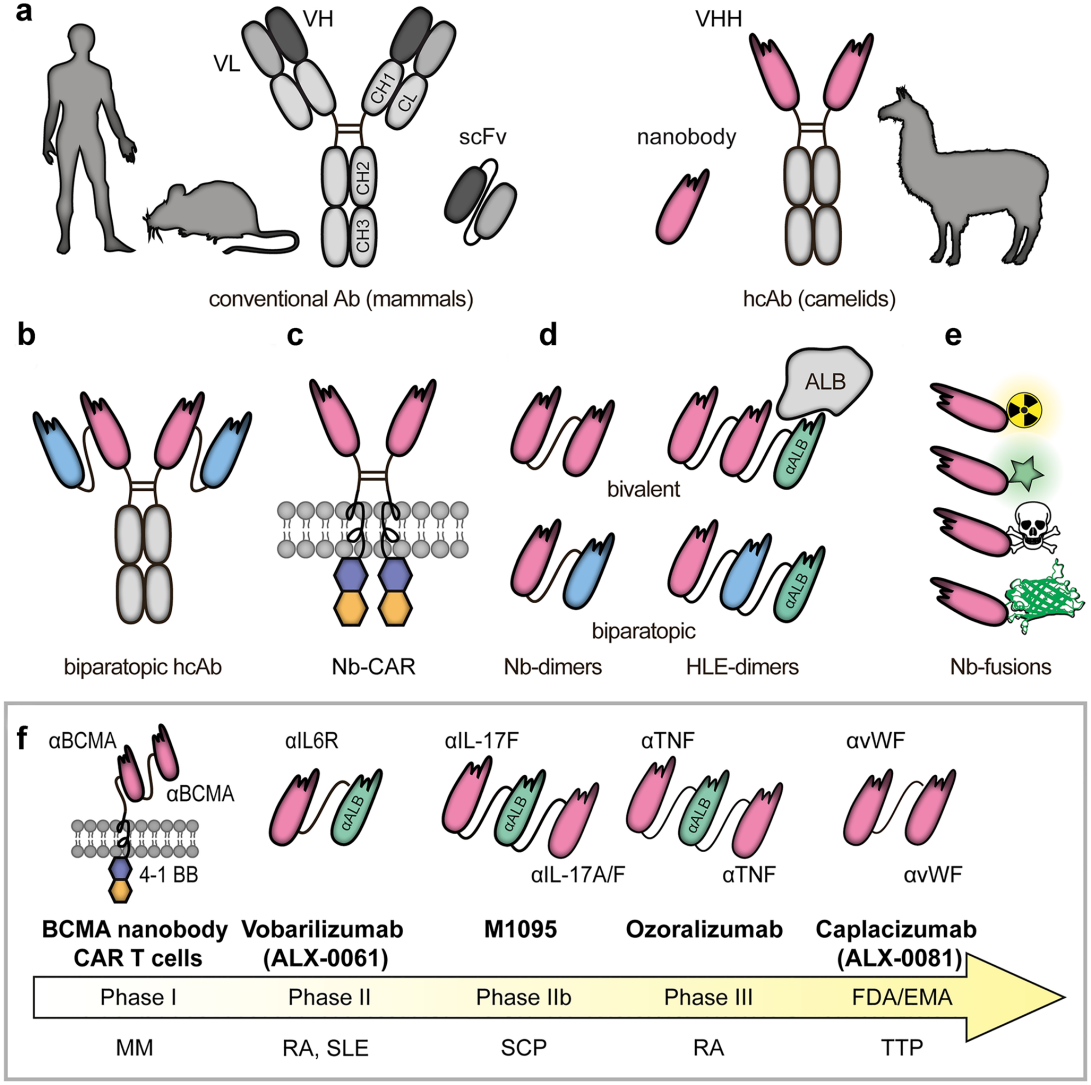
Structure, formats, and applications of VHHs. Conventional antibodies consist of two heavy and two light chains, and their antigen-binding region (paratope) is encoded by the variable domains of both chains (VH and VL). In case of camelid heavy chain antibodies, the antigen is recognized by the variable domain of the heavy chain (VHH) (**A**). Monomeric or dimeric (bivalent or biparatopic) nanobodies can be linked to the constant region (Fc) of any isotype to mediate different effector functions (e.g., complement dependent cytotoxicity (CDC) or antibody-dependent cellular cytotoxicity (ADCC)) (Schriewer et al. 2020) (**B**). VHHs can also be used as binding domains of chimeric antigen receptors (Nb-CAR) (Hambach et al. 2020) (**C**). Due to their modular structure, nanobodies can function as building blocks in multimeric constructs binding the same (multivalent) or different (multiparatopic) epitopes. The in vivo half-life of mono or multivalent nanobodies can be tuned, e.g., by genetic fusion to an albumin-specific nanobody (Tijink et al. 2008) (**D**). Monovalent VHHs can be conjugated chemically to radioisotopes (Huang et al. 2008) or fluorochromes (Fumey et al. 2017) and genetically to toxins (Mutter et al. 2018) and fluorescent proteins (Rothbauer et al. 2006) (**E**). Examples for nanobodies currently undergoing clinical trials include BCMA nanobody CAR T cells, Vobarilizumab, M1095, and Ozoralizumab, while Caplacizumab has already been FDA/EMA approved (**F**). scFv single-chain variable fragment, Ab antibody, hcAb heavy-chain antibody, ALB albumin, Nb nanobody, BCMA B cell maturation antigen, MM multiple myeloma, RA rheumatoid arthritis, SLE Systemic Lupus Erythematosus, SCP severe chronic psoriasis, TTP thrombotic thrombocytopenic purpura

Nanobodies are derived from naturally occurring heavy chain antibodies. These unusual immunoglobulin molecules were first detected in the serum of a dromedary in the early 1990s (Hamers-Casterman et al. 1993). In addition to heterotetrameric conventional antibodies, llamas and other camelids (camels, dromedaries, alpacas, vicuñas, guanacos) produce homodimeric antibodies consisting of only two heavy chains (Fig. 1).

The molecular basis for the generation of heavy chain antibodies in camelids can be explained by the missing CH1 domain of the heavy chain in two of the IgG-isotypes (Muyldermans et al. 1994) (Fig. 1A). Since the CH1 domain plays a major role in linking heavy and light chains, camelids can produce IgG isotypes consisting of only two heavy chains. In this case, antigen recognition is only performed by the variable domain of the heavy chain. Interestingly, camelids possess two subsets of variable domains that preferentially pair either with heavy chain or with conventional antibodies. The single variable domain of heavy chain antibodies (referred to as VHH or nanobody) is considered to be the smallest naturally occurring antigen-recognizing domain produced by the adaptive immune system (Muyldermans 2013).

Nanobodies have unique properties. With their long CDR3 regions, they can penetrate into functional cavities of proteins that cannot or only poorly be reached by the comparatively planar paratope of conventional antibodies (De Genst et al. 2006; Jahnichen et al. 2010; Maussang et al. 2013). Since functional clefts on proteins often correspond to the active site of an enzyme or the ligand binding pocket of a receptor, many nanobodies are excellent functional antagonists, an important property rarely attainable with conventional antibodies (Danquah et al. 2016).

Nanobodies have a number of other advantages over the variable domains of conventional antibodies, such as higher stability and solubility as well as better tissue penetration, reaching cell surface molecules in almost all major organs within minutes upon i.v. injection (Bannas et al. 2015; Bannas et al. 2014; Cheloha et al. 2020; Ingram et al. 2017; Rashidian et al. 2015). The high stability of VHHs is due to their structure consisting of two ß-folded sheets with a total of 9 ß-strands. The two ß-sheets are connected by a conserved canonical disulfide bridge connecting cysteine residues in framework (FR) 1 and 3. Some VHHs also have a second disulfide bridge linking the CDR3 with the CDR1 (lamas) or the CDR2 (camels) (Muyldermans et al. 1994).

Nanobodies are highly soluble and, in contrast to conventional VH domains, do not tend to aggregate due to hydrophilic amino acids in the FR2. The polarity of these amino acids promotes water solubility and also reduces the stickiness of the VHH domain. The corresponding FR2 region of the VH domain of conventional antibodies contains hydrophobic amino acids that mediate the association with the VL domain of the light chain (Wesolowski et al. 2009).

The modular structure of VHHs allows easy conversion into multivalent formats and easy linkage to functional groups or proteins (Fig. 1B–E). Importantly, the in vivo half-life of mono or multivalent nanobodies can be tuned, e.g., by genetic fusion to an albumin-specific nanobody (Tijink et al. 2008) (Fig. 1D). Similarly, genetic fusion to an engineered Fc-domain can endow nanobodies with potent effector functions, e.g., enhanced complement-dependent and antibody dependent cellular cytotoxicity (CDC, ADCC) (Schutze et al. 2018). These extraordinary features underline the high potential for the use of nanobodies in different therapeutic applications.

## Nanobodies in clinical trials

The physicochemical advantages of nanobodies over monoclonal antibodies (mAbs) have spurred the development of therapeutic nanobodies, some of which have entered clinical trials (Jovcevska and Muyldermans 2020) (Fig. 1F). As of April 2021, 13 nanobody drugs are undergoing phase 1–3 trials, either for oncologic or inflammatory diseases. The first therapeutic nanobody, caplacizumab (ALX-0081, Ablynx), was approved in the European Union in 2018 and in the USA in 2019 for the treatment of adults with acquired thrombotic thrombocytopenic purpura (aTTP) in conjunction with plasma exchange and immunosuppression. Caplacizumab is a humanized, bivalent von Willebrand factor (vWF)-specific nanobody dimer, which inhibits the interaction between vWF multimers and platelets, thereby preventing thrombocytopenia, hemolytic anemia, and tissue ischemia (Scully et al. 2019). Another promising nanobody (vobarilizumab/ALX-0061, Ablynx), currently in a phase II trial for treatment of Rheumatoid Arthritis (RA) and Systemic Lupus Erythematosus (SLE), is directed against the Interleukin-6 Receptor (IL-6R) (Van Roy et al. 2015). It consists of two nanobodies: one directed against IL-6R in order to inhibit the pro-inflammatory activities of the IL-6 pathway and the other is designed to extend the half-life of the therapeutic by binding to human serum albumin (HSA). A third example, a trivalent, interleukin-17-specific nanobody (M1095, Avillion LLP) is currently under development for the treatment of Severe Psoriasis. M1095 comprises three sequence-optimized, monovalent nanobodies that recognize IL-17A, IL-17F, and HSA (for half-life extension). M1095 possesses humanized sequences to reduce immunogenicity. With three immunoglobulin domains, it is considerably smaller than conventional IgG antibodies with 12-Ig domains (40 kDa vs. 150 kDa) (Svecova et al. 2019).

Nanobodies also have advantages compared to conventional single-chain variable fragments (scFv) in the design of chimeric antigen receptors (CAR) (Fig. 1A, C). To direct T cells against oncologic targets, they are genetically modified to express CARs consisting of an extracellular scFv or nanobody domain connected via a transmembrane domain to intracellular signaling modules derived from other cell surface signaling proteins (June et al. 2018). CAR T cell therapy is currently a rapidly developing approach for targeted therapy of specific cancer entities (Sermer and Brentjens 2019). It has proven successful in the treatment of hematological cancers but concerns remain about adverse effects. Furthermore, the efficiency to target solid tumors is still limited, and there is still a need for improved and safe alternatives (Xie et al. 2019). Nanobody CAR T cells may help to increase the local inflammatory response for improved immune recognition and enhance drug access to the tumor (Xie et al. 2019). Nanobody CAR T cells currently in active trials include BCMA nanobody CAR T cells against multiple myeloma, αPD1-MSLN-CAR T cells for colorectal and ovarian cancer, and CD19/CD20 bispecific CAR T cells against B-cell lymphoma (https://clinicaltrials.gov) (Jovcevska and Muyldermans 2020).

Nanobodies are presently also under investigation as potential therapeutics against SARS-CoV-2 infection (Dong et al. 2020; Konwarh 2020). Nanobodies are aimed against the receptor binding domain (RBD) of the spike protein of SARS-Cov-2 to prevent binding of the virus to the ACE2 receptor on host cells. Studies have identified two closely related nanobodies, H11-D4 and H11-H4, that bind RBD and block its interaction with ACE2. Nanobody-Fc fusion proteins showed neutralizing activity against SARS-CoV-2 and additive neutralization with the SARS-CoV-1/2 antibody CR3022 pointing to a possible therapeutic exploitation for the development of an inhalable drug as a prophylaxis against COVID-19 (Dong et al. 2020; Huo et al. 2020; Konwarh 2020).

## The kidney’s role in nanobody applications

Due to their small size and modular structure, nanobodies offer advantages for various applications. However, with regard to renal retention, the small size of nanobodies in combination with the use of peptide tags also brings difficulties for some applications. Since the size of nanobodies is well below the filtration threshold of the glomerular membrane, nanobodies are rapidly excreted in the urine after their application (Bannas et al. 2015; Bannas et al. 2014; Debie et al. 2020). This is advantageous for molecular imaging of structures that are not close to the kidneys, as background signals decrease very rapidly and toxic side effects are minimized (Vaneycken et al. 2011). Depending on the format in which the nanobodies are applied, they may be retained in the kidney. For targeting structures close to the kidney, renal retention of nanobodies is disadvantageous because the strong signal in the kidney makes specific staining of adjacent tissue difficult (Bao et al. 2021). It has been shown that renal retention of nanobodies is primarily determined by the number of polar residues in the C-terminal amino acid tag. For a radiolabeled HER2-specific nanobody, application in the untagged format resulted in a decreased renal accumulation of almost 90% compared to a Myc-His-tagged format (D’Huyvetter et al. 2014).

Additional strategies have been developed to further reduce the accumulation of nanobodies in the kidney. Renal retention occurs through the endocytic apparatus of the proximal renal tubule. In this complex of different molecules, megalin is responsible for at least 40% of the total protein retention in the kidneys. Simultaneous injection of substances such as gelofusine, lysine, or monosodium glutamate can reduce renal reabsorption of nanobodies by inhibiting their binding to megalin (D’Huyvetter et al. 2014; Gainkam et al. 2011; Rousseau et al. 2018).

## Nanobodies in kidney disease

Renal diseases comprise primary kidney disorders, such as diseases of the glomerulus (nephrotic syndrome or glomerulonephritis), the tubulointerstitium, or the reaction of the kidney against toxins (viral toxins, myoglobin), drugs (nonsteroidal anti-inflammatory drug), or diagnostic agents (contrast dyes, antibiotics), as well as infections and renal neoplasia (Basile et al. 2012). Additionally, kidney damage can result from many systemic diseases, such as heart failure, hypertension, and diabetes (Kazancioglu 2013). Apart from management of hypertension and treatment of the underlying conditions, therapeutic options are still limited. Patients with a declining renal function often progress to end stage renal disease (ESRD), which requires dialysis and renal transplantation (Krolewski et al. 2017). World-wide, patient numbers with chronic kidney disease and ESRD are on the rise, placing a huge burden on societies and health care systems (Hill et al. 2016; Szczech and Lazar 2004). Due to the limited treatment options and slow progress in recent decades for kidney-specific medications, the need for novel therapeutics remains high. Therefore, new avenues have to be explored to find novel drugs and biologics for the treatment of renal diseases. In many aspects of renal diseases, e.g., imaging, diagnostics and therapy, nanobodies might prove beneficial.

### Glomerular diseases

The glomerular filter consists of 3 layers: fenestrated endothelium, glomerular basement membrane, and podocytes. The latter builds a complex interdigitated net around the capillary loops with the secondary foot processes forming the slit diaphragm, the smallest part of the glomerular filter. While large proteins are not able to pass the glomerular filter, antibodies targeting podocyte transmembrane proteins are known to cross the glomerular basement membrane (Akilesh et al. 2008). Thus, we presume that this is also true for the much smaller (half-life extended) nanobodies. Stabilization of the slit diaphragm has been implicated in protection against podocyte foot process effacement and proteinuria (Kawachi and Fukusumi 2020). Thus, enhancement of Neph1 - ZO-1 interaction was shown to protect from injury-induced renal damage (Sagar et al. 2017). Furthermore, several surface molecules on podocytes have been shown to be involved in glomerular diseases. Up-regulation of HB-EGF has been shown to lead to activation of EGFR, and rapid progressive glomerulonephritis (RPGN) can be attenuated by EGFR deletion in podocytes or by pharmacological blockade (Bollee et al. 2011). EGFR-specific nanobodies are well characterized for the treatment and detection of tumors and have been used in various formats (Roovers et al. 2011; Sharifi et al. 2021; Tintelnot et al. 2019). Additionally, it is conceivably that nanobody-mediated blockade of autoantibody binding to podocyte membrane proteins such as THSD7A and PLA2R might prove a useful therapeutic measure in treatment of membranous nephropathy (Beck et al. 2009; Tomas et al. 2014). Furthermore, blocking circulating molecules, such as suPAR, via nanobodies from inducing harm on kidney cells harbors therapeutic potential in glomerular diseases (Wei et al. 2011; Zeier and Reiser 2017). Besides podocytes, surface proteins of parietal epithelial cells surrounding the glomeruli, such as CD9, also represent potential novel nanobody targets, e.g., to treat or prevent glomerulosclerosis (Lazareth et al. 2019).

A hallmark of the glomerular damage is proteinuria caused by a leaky glomerular filter. Compromised filtration is bound to also affect nanobody half-life. This might require increasing the frequency of the nanobody administration during proteinuria. In contrast, with decreasing glomerular filtration rate (GFR) in kidney disease, accumulation of nanobodies in the kidney is a conceivable issue. Here, decreased blood flow and drainage by the lymphatic system might act as counteracting forces. The pharmacodynamics of nanobody treatments specifically for kidney diseases will have to be explored in future studies.

### Acute kidney injury

Acute kidney injury (AKI) is reported in more than 50% of intensive care patients and involves clinical syndromes with acute renal dysfunction caused by sepsis, drug toxicity, and ischemia reperfusion injury (Gao et al. 2020; Ronco et al. 2019). Patients have a high mortality and risk to develop chronic kidney disease (CKD) (Coca et al. 2012; Hsu 2012). Several growth factors have been implicated in either attenuating or aggravating AKI. EGF administration and EFR activation appear to promote recovery of renal function after ischemia-reperfusion injury. However, their roles in fibrosis indicate that they may be unfit as targets unsuitable for long-term treatment (He et al. 2013; Humes et al. 1989; Tang et al. 2013). Of the multiple fibroblast growth factors with known roles in AKI, FGF-23 and its co-receptor Klotho have been implicated as potential predictive and prognostic biomarkers for AKI (Christov et al. 2019). As Klotho expression decreases with increasing kidney damage, stabilization of Klotho via nanobodies would represent a promising approach for individualized patient treatment (Ray et al. 2020). Likewise, bFGF and its receptor bFGFR2 have been shown to promote kidney recovery; thus, activation of bFGFR2 might have therapeutic potential (Villanueva et al. 2006; Villanueva et al. 2008). Tgfbr2 deletion in macrophages was shown to ameliorate AKI, indicating TgfβrII as a potential target to prevent tubulointerstitial fibrosis after severe ischemic renal injury (Chung et al. 2018). Another signaling axis, podocyte expression of VEGF, such as VEGF-121, and its signaling via VEGFR2 has been shown to be renoprotective in tubulointerstitial diseases, but not in diabetes (Facemire et al. 2009; Leonard et al. 2008). While homeostasis, milieu, isoform, site, and mode of action seem of vital importance for achieving beneficial effects, targeting this pathway with nanobodies is worth considering (Majumder and Advani 2017).

### Inflammatory kidney disease

Promising advances have also been made with mAbs in inflammatory renal diseases, for which the standard protocols have for decades been based only on corticosteroids and non-specific immunosuppressants with heavy side effects for the patients (Santoro et al. 2015). Important mAbs for the treatment of inflammatory kidney disorders include rituximab (used in membranous glomerulonephritis, steroid-resistant nephrotic syndromes and anti-neutrophil cytoplasmic antibodies (ANCA)-associated vasculitis, membranoproliferative glomerulonephritis (MPGN)), eculizumab (used in typical hemolytic uremic syndrome, C3 nephropathy and MPGN), and fresolimumab (focal segmental glomerulosclerosis and kidney cancer) (Santoro et al. 2015). In one recent approach, anti-inflammatory nanobodies have targeted the adenosine 5′-triphosphate (ATP)–gated P2X7 ion channel (Danquah et al. 2016)**,** (Menzel et al. 2018). P2X7 is expressed on macrophages and CD4-positive T cell subpopulations. Upon sensing ATP released as a danger signal from cells during infection and sterile inflammation, P2X7 initiates a proinflammatory signaling cascade resulting in the release of the pro inflammatory cytokines IL-1β and IL-18. Treatment with the P2X7-blocking nanobody 13A7 ameliorated disease in mouse models of glomerulonephritis and contact dermatitis, while treatment with the P2X7-agonistic nanobody 14D5 aggravated glomerulonephritis (Danquah et al. 2016). As P2X7 constitutes a trimeric ion channel, both nanobodies had increased potency as dimers or Fc fusion proteins. Furthermore, Dano1, a nanobody generated against human P2X7, inhibited the release of IL-1β from endotoxin-treated human blood cells 1000-fold more potently than the small-molecule drugs against P2X7 currently in development (Danquah et al. 2016).

While anti-TNF treatment was so far not effective for maintenance of remission in ANCA-patients (Wegener’s Granulomatosis Etanercept Trial Research 2005), its receptors TNFR1 and TNFR2 might be potential targets for nanobodies in inflammatory kidney disease (Speeckaert et al. 2012). Targeted blockade of either TNFR1 or TNFR2 might be effective by preserving other proinflammatory and immunosuppressive functions (Ernandez and Mayadas 2009). So far, several nanobodies have already been developed against various chemokines, potentially enabling effective and specific treatment of renal inflammatory diseases by inhibiting chemokine receptor activation and chemotaxis (Blanchetot et al. 2013).

In autoimmune diseases affecting the kidney, such as membranous nephropathy or SLE, targeting autoantibody-producing plasma cells is a promising therapeutic strategy (Hofmann et al. 2018)**.** Among others, proteasome inhibitor bortezomib (Neubert et al. 2008; Tasaki et al. 2019) and CD20-specific monoclonal antibody rituximab (Uematsu-Uchida et al. 2019) have been successfully used. Here, using nanobodies in a heavy-chain format to induce antibody-dependent cellular cytotoxicity is a promising and specific targeting strategy to deplete autoantibody-producing cells. Several nanobodies targeting B cells and plasma cell have been produced so far for anti-tumorigenic purposes, but their effectiveness for depletion of autoantibodies in the kidney has yet to be investigated (Schriewer et al. 2020; Zhao et al. 2018).

### Renal cell carcinoma

In recent years, many mAbs have been approved for the treatment of cancers (Chiavenna et al. 2017). Due to their high specificity towards malignant cells, mAbs have distinct advantages over conservative therapies, such as chemotherapy, radiotherapy and surgery, and can act in synergy with these therapies. For example, bevazizumab (Avastin), an antiangiogenic VEGF-A-specific mAb, was approved in 2004 for the treatment of colon cancer. Avastin is also indicated for the treatment of metastatic renal cell carcinoma in combination with interferon alpha. Renal cell carcinoma is the most common type of kidney cancer in adults and has a poor prognosis in stage III and IV (Siegel et al. 2017; Znaor et al. 2015). In a phase III trial, bevazizumab treatment of the highly vascularized tumor led to a reduction in the risk of disease progression by 37% but no overall survival benefit (Escudier et al. 2007; Rini et al. 2010)**.** Anti-angiogenic therapy has since been combined with immunotherapy for better results (Garcia et al. 2020).

As nanobodies are able to adopt all the different targeting strategies of mAbs, such as direct targeting of cancer cells, cytotoxic moiety delivery, or modification of the host immune response, they have the potential to offer additional benefits. For instance, monomeric anti-Her2 nanobodies show a faster accumulation and more homogenous distribution in the tumor within minutes after injection in the mouse model than mAbs (Debie et al. 2020). Nanobodies could also be utilized in concert with the immune system to target cancer cells. One potential target is CD38, an ecto-enzyme that is highly expressed on the surface of malignant plasma cells in multiple myeloma (Lin et al. 2004; Mesguich et al. 2016). Here, nanobody-based CD38-specific hcAbs are able to induce antibody-dependent cellular cytotoxicity in tumor cancer cell lines and inhibit tumor growth in a mouse xenograft model, warranting further clinical development as therapeutics for multiple myeloma and other hematological malignancies (Schriewer et al. 2020). Furthermore, nanobodies can be conjugated to deliver drugs to the target cell as demonstrated by tetrameric anti-EGFR nanobodies (7D12) conjugated to tetravalent platinum Pt(iv) prodrugs (e.g., cisplatin) to generate a nanobody–drug conjugate (NDC) (Wu et al. 2020). In a mouse model, this NDC showed high targetability, high antitumor efficacy, and low systemic side-effects (Wu et al. 2020). Thus, nanobodies with their many possibilities for formatting, linking, and targeted drug-therapy may have potential for novel approaches for the diagnosis and treatment of renal cancer.

### Renal replacement therapy

Patients progressing to ESRD require renal replacement therapy, either dialysis or renal transplantation, as a last resort. Major issues with renal transplantation involve activation of the complement system, e.g., due to ischemia-reperfusion or antibody-mediated rejection. The C5-specific mAb eculizumab (Legendre et al. 2013) has been used in kidney transplantation to prevent delayed graft function, in antibody-mediated rejection, atypical hemolytic uremic syndrome (aHUS) recurrence, and anti-phospholipid syndrome (Legendre et al. 2017). While trials did not show efficacy in delayed graft function, a study did show a decrease in antibody-mediated rejection (Stegall et al. 2011). However, there was no long-term benefit of eculizumab-mediated C5 blockage in regard to transplant glomerulopathy and microvascular inflammation after 2 years (Cornell et al. 2015) and an early benefit is still debated. Likewise, C1 esterase inhibitors have been used in renal transplantation with variable results (Bhalla et al. 2020; Huang et al. 2020b; Montgomery et al. 2016). Nanobodies against members of the complement cascade, such as C5 and C4b, have shown promising selective inhibition (Yatime et al. 2018; Zarantonell et al. 2020). Complement-inhibiting nanobodies, perhaps in a half-life extended format, may have the potential for synergistic and/or more cost-effective treatment in renal replacement therapy. Furthermore, plasma cell depletion has also been used to counteract antibody-mediated graft rejection (Kwun et al. 2019; Tasaki et al. 2019), indicating a potential therapeutic strategy by use of nanobodies as discussed above.

In a dialysis setting, a recent proof-of-concept study showed that nanobodies are suitable immunosorbents to specifically purify toxic substances from the blood. In this specific example, a highly selective immunosorbent was prepared using a β2-microtubulin- (β2M-) specific nanobody to prospectively purify β2M from the blood of patients with dialysis-related amyloidosis (DRA). The accumulation of β2M is a serious side effect in patients with end-stage kidney disease, indicating that selective extracorporeal removal of β2M is a promising method to delay the onset and progression of DRA (Huang et al. 2020a).

### Imaging and diagnostics

Due to their small size and excellent tissue penetration properties, nanobodies have been successfully used for in vivo imaging. Optical imaging is achieved by combining nanobodies with near-infrared (700–900 nm) fluorophores, such as AlexaFluor680, Cy7, or quantum dots (Qd800) (Fatehi et al. 2014; Zheng et al. 2019). This was used, for example, in an experimental mouse models as intraoperative fluorescence imaging to increase the sensitivity of peritoneal tumor implant debulking surgery (Debie et al. 2018). Comparisons of nanobodies, heavy-chain antibodies, and conventional antibodies labeled with AlexaFluor680 showed that monovalent nanobodies had the best signal-to-noise ratio due to the lowest background staining, while the heavy-chain antibody showed the highest labelling efficiency (Bannas et al. 2014).

Deeper tissue penetration can be achieved by combining radionuclide-based techniques such as SPECT (single-photon emission computed tomography; e.g., ^99m^Tc or ^111^In) (Gomes et al. 2011) and PET (Positron emission tomography, e.g., ^18^F, ^64^Cu, ^68^Ga, or ^89^Zr) (Ametamey et al. 2008; Chakravarty et al. 2014) with nanobodies**,** which has been successfully used in mouse models of cancer or atherosclerosis (Balhuizen et al. 2017; Jailkhani et al. 2019; Keyaerts et al. 2016). In a Phase I study of ^68^Ga-labelled HER2 nanobody for PET/CT in breast carcinoma, the treatment showed no adverse reactions and a fast blood clearance of 90% within 1 h with a radiation dose comparable to other routinely used PET tracers (Keyaerts et al. 2016). Experimental studies are also focusing on imaging of inflammatory diseases with ^99m^Tc, such as nuclear imaging of atherosclerotic lesions or tumor-associated macrophages (Broisat et al. 2012; Movahedi et al. 2012; Schoonooghe et al. 2012).

Going even further, the diagnostic potential of nanobodies can be linked to therapeutic properties. For instance, nanobody-photosensitizer conjugates, such as IRDye700DX, are able to induce cell toxicity upon treatment with near-infrared light called photo dynamic therapy and trigger immune responses (Beltran Hernandez et al. 2020; De Groof et al. 2019). Furthermore, novel ultrasound molecular probes using nanobody-coupled lipid nanobubbles for enhanced ultrasound imaging of renal cell carcinoma or prostate cancer are potential carriers of therapeutics, which can be released through an ultrasound-targeted nanobubble destruction technique for local accumulation of the drug (Fan et al. 2015; Yu et al. 2020)**.** Many more applications for the kidney are imaginable in terms of imaging and diagnostics, e.g., analysis of renal fibrosis, perfusion, and filtration.

## Summary and conclusion

**Fig. 2:**
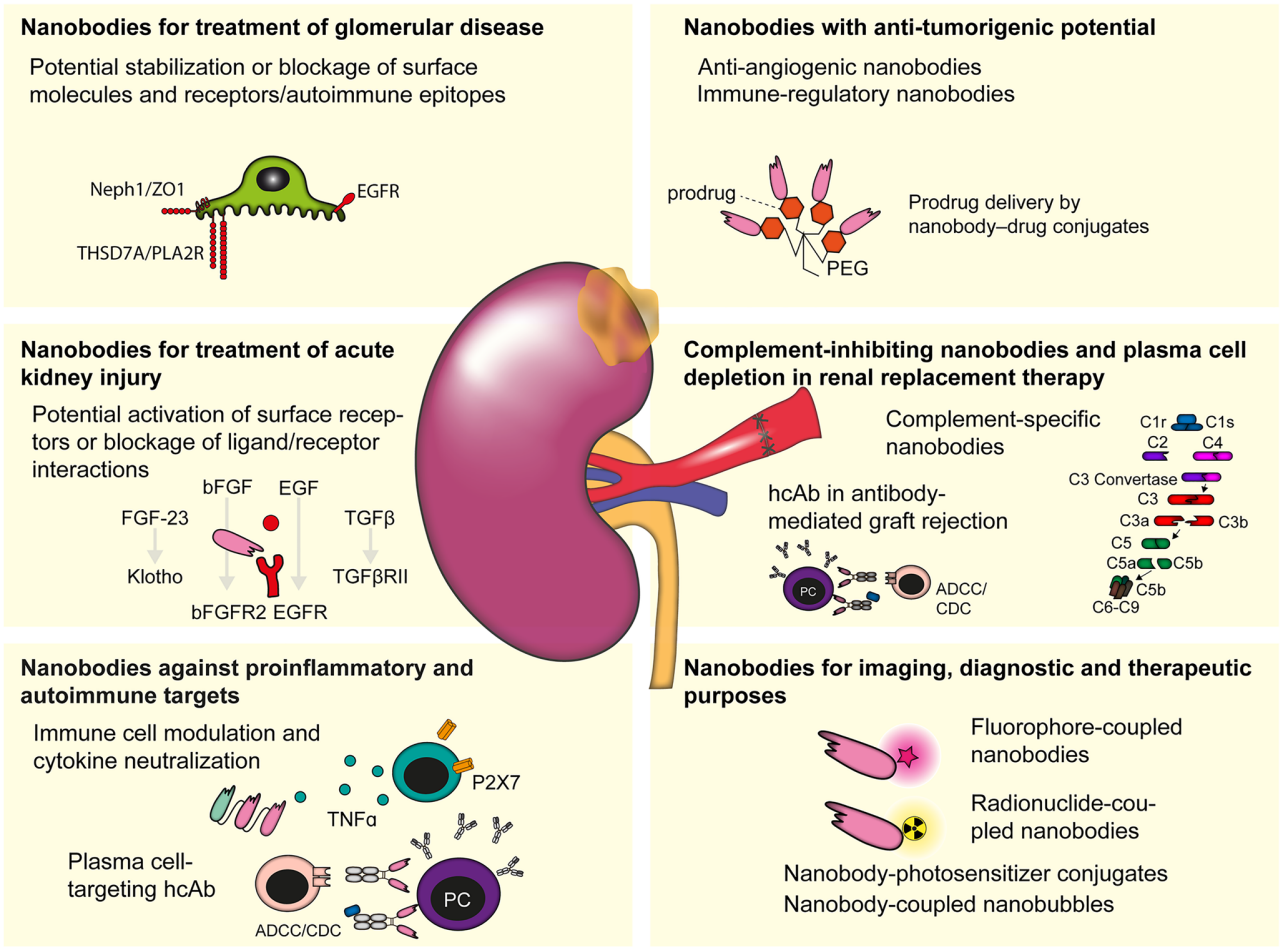
Potential applications for nanobodies in kidney disease. PEG polyethylene glycol, PC plasma cell, CDC complement-dependent cytotoxicity, ADCC antibody-dependent cellular cytotoxicity

Camelid VHH domains have been tailored for expression as heavy chain antibodies throughout 50 million years of evolutionary history. The resulting biochemical properties distinguish them from human VH domains: small size, excellent solubility, good tissue penetration, and rapid elimination via the kidney combined with the possibility of targeted conjugation of dyes make nanobodies particularly interesting for use in imaging applications. Moreover, numerous clinical and preclinical studies have already demonstrated that nanobodies can be used as molecular building blocks for highly efficient drugs in various therapeutic applications. The approval of the first therapeutic nanobody (caplacizumab) is expected to pave the way for further nanobody-based products in immunotherapy. Due to the limited treatment options, there is a huge unmet need in nephrology for novel and specific therapies. Thus, nephrology could profit immensely from tailored nanobody applications, e.g., by stabilizing the filtration apparatus, by neutralizing pro-inflammatory cytokines, complement components, and purinergic receptors, by blocking the binding of fibrosis-promoting growth factors, by blocking the binding of autoantibodies, or for the extracorporal removal of nephrotoxic proteins from the blood (Fig. 2). In the future, enhanced disease stratification will lead to a better understanding of disease entities and increasingly individualized medicine which make utilization of nanobodies excellent for the development of novel innovative treatments for kidney diseases and beyond.
